# A lateral flow strip based on gold nanoparticles to detect 6-monoacetylmorphine in oral fluid

**DOI:** 10.1098/rsos.180288

**Published:** 2018-06-27

**Authors:** Jia Liu, Xiaolong Hu, Fangqi Cao, Yurong Zhang, Jianzhong Lu, Libo Zeng

**Affiliations:** 1 School of Pharmacy, Fudan University, Shanghai 200433, People's Republic of China; 2 Shanghai Institute of Pharmaceutical Industry, Shanghai 200437, People's Republic of China; 3 Shanghai Key Laboratory of Crime Scene Evidence, Shanghai Institute of Forensic Science, Shanghai 200083, People's Republic of China; 4 Shanghai Key Laboratory of Crime Scene Evidence, Shanghai Research Institute of Criminal Science and Technology, Shanghai, People's Republic of China

**Keywords:** gold nanoparticles, heroin, 6-monoacetylmorphine, morphine, oral fluid, biosynthesis

## Abstract

We used lateral flow strips based on gold nanoparticles to detect 6-monoacetylmorphine (6-MAM; heroin's unique metabolite) in oral fluid samples. In this competitive lateral chromatographic immunoassay, the 6-MAM was chemically synthesized and conjugated to bovine serum albumin. The results were qualitatively detected via the colour change of the test line. By using a proper sample pad, a suitable nitrocellulose membrane and a customized sponge device adsorbed the oral fluid directly from the mouth; the total test time was 3 min. The sensitivity of the assay was 4.0 ng ml^−1^ without any cross-reactivity with 10 normal drugs, which are widely subject to abuse, including morphine and codeine. This test could be easily used on site to detect heroin in oral fluid, and it could be a promising product in the future including for driving under the influence.

## 1. Introduction

Opioid abuse has risen dramatically in recent years and is a major cause of morbidity and mortality. According to the World Drug Report 2017 published by the United Nations Office on Drugs and Crime, the use of opiates and prescription opioids may not be as widespread as cannabis, but opioids remain major drugs with potential harm and health consequences [1]. Therefore, the facile and rapid detection of opioids is an urgent need.

**Figure 1.**
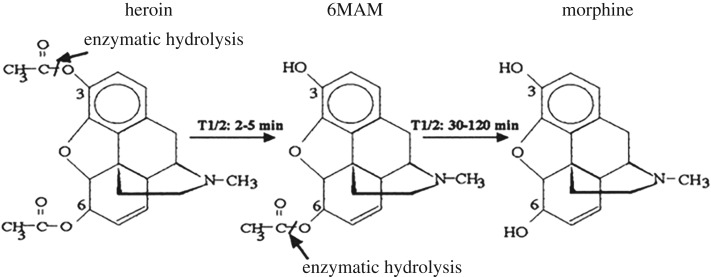
Heroin hydrolysis and *in vivo* metabolism. The structures of heroin, 6-MAM and morphine are shown.

Illicit heroin abuse is one of the most common forms of opioid addiction. Heroin (diacetylmorphine, diamorphine or Diagesil®) is a semi-synthetic morphine derivative and a powerful opioid analgesic [2]. The metabolism of heroin is visualized in figure 1 [3]. Heroin rapidly hydrolyses to 6-monoacetylmorphine (6-MAM) and finally into morphine. As heroin hydrolyses quickly after administration, its metabolites are usually employed to confirm use. Furthermore, 6-MAM is the only specific indicator of recent heroin abuse versus morphine, and it has aroused great interest from the research community.

Several methods to detect 6-MAM have been described, and these can be divided into the following categories: (1) chromatographic analysis including gas chromatography [4–7] and high-performance liquid chromatography [8–11]; (2) spectroscopic analysis such as Ramen spectroscopy, infrared spectroscopy, chemiluminescence [12], etc.; (3) capillary electrophoresis [13–15]; and (4) immunoassay methods (antigen–antibody) [16–18]. Complex instrumentation techniques place tremendous pressure on basic drug screening because of the need for sophisticated equipment and professional operators. The laboratory is sometimes closed or far away. Not even police stations can house this complex and expensive equipment. However, a police officer has to judge immediately whether a suspected material contains heroin or not and needs to react swiftly. Thus, the development of specific, reliable and simple methods to detect illicit drugs in biological samples is urgently needed [19].

Of the methods for rapid detection, colloidal gold nanoparticle (AuNP)-based lateral flow strips (LFSs) have been widely adopted for rapid screening due to the size-dependent and distance-dependent optical property of AuNPs, with the first report by Mirkin and co-workers [20]. The principle of semi-quantitative lateral flow assays is that the red colour of the AuNPs can be viewed by the naked eye from the antigen–antibody combination in several minutes [21–23]. Various commercial test kits for heroin abuse detection are available including those from the NovaBios and Wondfo companies. However, most heroin screening kits only measure morphine but not 6-MAM, because it is difficult to discriminate between 6-MAM and morphine. Morphine could be metabolized from other drugs or could have been prescribed. The 6-MAM is uniquely traceable to heroin.

Samples include blood, plasma, urine, hair, oral fluids as well as in breath, sweat, breast milk, teeth, etc. [24–27]. The most common samples used for illicit heroin testing are blood, urine and oral fluids. Of these, the blood test is the most accurate and reliable, but it is also invasive. Urine testing is the most convenient and widely used in drug of abuse screening. Oral fluids are increasingly used for point of care testing—they are easy to collect in public. However, oral fluids are very viscous and have low concentrations of target; thus, most tests use urine for 6-MAM testing. All oral fluid test development will face the same problem for sample collection and sensitivity improvement. A previous study [28–30] showed that 6-MAM is frequently detected in oral fluid. The detection standard of LFSs in oral fluid 6-MAM is 4 ng ml^−1^ [31,32]. Here, we developed a lateral flow test for heroin in oral fluid samples.

We explored AuNPs as antibody labels in a lateral flow assay for rapid and sensitive detection of 6-MAM via a colorimetric signal. First, we synthesized the 6-MAM and then conjugated it to bovine serum albumin (BSA) to enable it to be coated on a T line. Moreover, to overcome the difficulties in dealing with oral fluid samples, the types of nitrocellulose (NC) membranes, the solution formula of the sample pad and the sponge adsorbent pad were chosen to search the best conditions for oral fluid LFSs. Finally, the 6-MAM LFS was validated and shown to have outstanding sensitivity and specificity.

## 2. Experimental

### 2.1. Materials

The antibodies against 6-MAM were supplied by Bioventure (Shanghai). BSA and polyvinyl pyrrolidone (PVP) were purchased from Sigma (Barcelona, Spain). Triton X-100, Tetronic 1307 (S9), Ohodasurf On-870 (S17) and STANDAPOL ES-1 (S7) were purchased from BASF (Germany). Distilled water (resistivity 18.2 MΩ cm^−1^) was made by a RephiLe PURIST UV Ultrapure water system (China). The Reel dispersion system was from Doyesgo (China). The Vion IMS Q-Tof mass spectrometer was from Waters (USA). All standard materials such as 6-MAM and morphine were obtained from the National Institutes for Food and Drug Control (China). The microscope was from Motic AE2000 (Xiamen, China). All other chemical and immunological reagents not specified here were standard commercial products of analytical/reagent grade.

### 2.2. The components of the lateral flow strip

**Figure 2.**
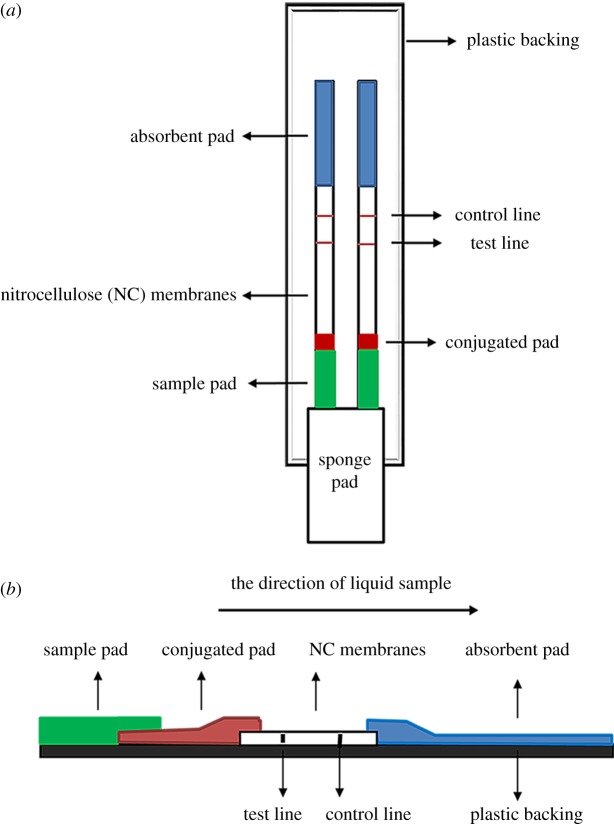
Schematic view of the lateral flow strip. (*a*) Vertical view of the lateral flow strip. (*b*) Side view of the lateral flow strip.

**Figure 3.**
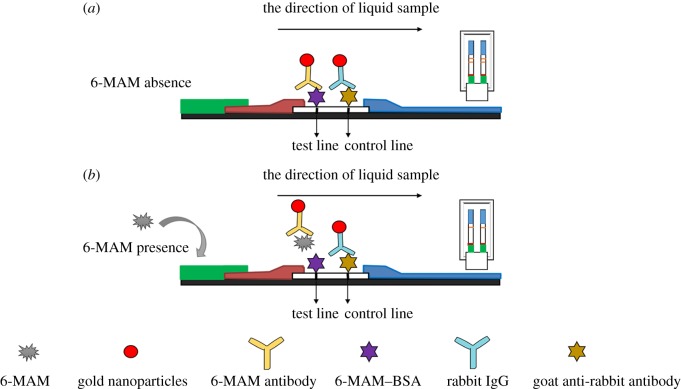
Schematic diagram of the lateral flow strip for 6-MAM detection. (*a*) 6-MAM is absent. (*b*) 6-MAM is present.

The LFSs consist of a plastic backing, a sponge adsorbent strip (sponge pad), a sample pad, a conjugated pad, NC membranes and an absorbent pad. The sponge adsorbent strip is specially designed for oral fluid collection and quickly transports oral fluid into the sample pad. The sample pad contains a buffer system and some surfactants. The antibody–AuNP conjugates were sprayed on the conjugate pad to react with sample and be released from the pad and enter the NC membrane coated with 6-MAM–BSA on the T line and goat anti-rabbit antibodies on the C line. The absorbent pad is a filter paper located at the end of the strip; it maintains the capillary flow. The LFS only needs to be placed into the mouth or inserted into an oral fluid sample cup. A schematic view of the LFS is shown in figure 2. A schematic diagram of the LFS for 6-MAM detection is exhibited in figure 3.

### 2.3. Synthesis of 6-monoacetylmorphine–bovine serum albumin conjugate

**Figure 4.**
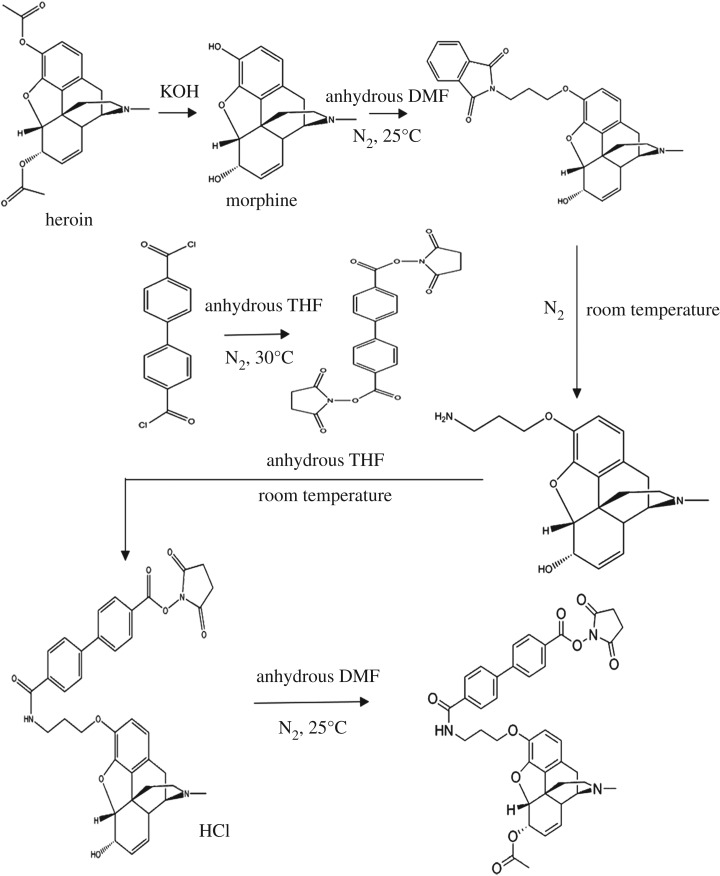
A chemical reaction pathway of the preparation of activated 6-MAM.

**Figure 5.**
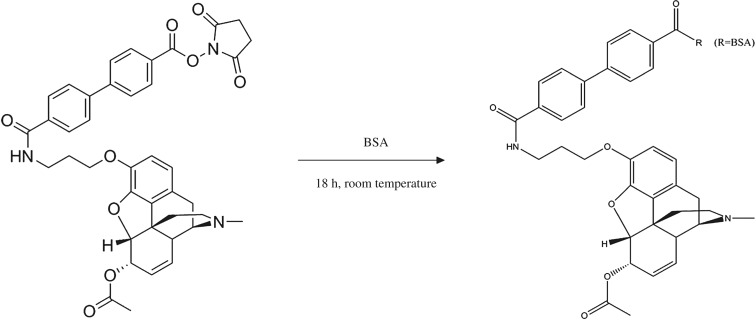
A chemical reaction pathway of the preparation of the 6-MAM–BSA conjugate.

The 6-MAM was prepared as described previously in research works [16]. Briefly, morphine was first produced by heroin alkali hydrolysis. An *N*-hydroxysuccinimide (NHS) ester group was then added to the 6-MAM molecule to conjugate it to the carrier proteins (figure 4). The activated 6-MAM was assured by a Waters® Vion IMS Q-Tof mass spectrometer. Next, the synthesis was performed as described (figure 5) with some modifications. First, 80 mg of BSA in 6 ml of 50 mM potassium phosphate buffer (pH = 7.5) was allowed to cool to 0°C. Then, 20 mg activated 6-MAM in 1 ml of anhydrous dimethylformamide (DMF) was added dropwise at 0°C. The mixture was warmed to room temperature and stirred overnight. The resulting 6-MAM–BSA conjugate was dialysed against 50 mM potassium phosphate buffer (pH = 7.5) with six buffer changes (at least 6 h each at 4°C).

### 2.4. Preparation of gold nanoparticles–antibody conjugates

The 20 nm AuNPs were prepared via a citrate reduction method [33]. Here, 2 ml of 1% HAuCl_4_ solution was added into 100 ml boiling water with vigorous stirring, and then 2 ml of 1% sodium citrate solution was immediately added. When the solution turned red, it was boiled for another 15 min. The solution was cooled to room temperature and stored at 4°C for further use.

After adjusting the pH value of the AuNPs solution to 9.0 by 0.1 M K_2_CO_3_, 30 µg of 6-MAM antibodies was added into 10 ml of AuNPs solution and incubated for 30 min. This was followed by 20 µl of 100.0 g l^−1^ BSA for 15 min to block reactive sites. The solution was centrifuged at 3740*g* for 15 min, and the supernatant was re-centrifuged at 12 100*g* for another 30 min. All gold precipitates were mixed and measured to identify the maximum absorbance via UV–visible spectroscopy. It was then stored at 4°C for further use.

The same method was used to conjugate AuNPs to rabbit IgG antibodies. When making the conjugate pad, the antibody conjugates were diluted to absorbance five with buffer (0.05 M Tris-HCl containing 10.0 g l^−1^ BSA, 0.4% Triton X-100, 5% trehalose, 10% sucrose, pH 8.2). Finally, 500 µl of mixed AuNPs–antibody conjugates were sprayed on a 20 mm^2^ fibreglass and then dried at 37°C overnight.

### 2.5. Preparation of coated nitrocellulose membrane

To make the lateral flow test strip, 6-MAM–BSA antigens (0.6 mg ml^−1^) were dispensed on the NC membranes as the test lines (T line). Control lines (C line) were coated by goat anti-rabbit polyclonal antibodies (0.15 mg ml^−1^). The coated NC membranes were dried at 37°C overnight. Nine commercial NC membranes from four companies were evaluated: Millipore (HF90, HF135 and HF180), GE-Whatman (FF120HP and AE100), Sartorius (CN95 and CN150) and Pall (Vivid90 and Vivid170).

### 2.6. Sensitivity and specificity

The strip is a competitive assay, and both positions had 6-MAM strips. When the sample contains 6-MAM, it binds to the nanogold-labelled antibody on the conjugated pad. Excess antibodies continue to advance along the chromatographic direction due to capillary action and then bind to the 6-MAM antigen on the T line. The signal intensity of the T line is directly related to the 6-MAM concentration in the sample. A darker colour indicates a lower 6-MAM concentration.

Negative oral fluid was collected from six people and spiked with 6-MAM (400, 100, 40, 10, 4, 1, 0.4, 0.1 ng ml^−1^) for sensitivity detection. Ten commonly abused drugs were used to verify the specificity of the LFSs. These drugs were morphine (MOP, 100 µg ml^−1^), codeine (COD, 100 µg ml^−1^), tetrahydrocannabinol (THC, 10 µg ml^−1^), methylene dioxymethamphetamine (MDMA, 100 µg ml^−1^), ketamine (KET, 100 µg ml^−1^), methylamphetamine (MET, 100 µg ml^−1^), cocaine (COC, 100 µg ml^−1^), methadone (MTD, 100 µg ml^−1^), ephedrine (EPH, 100 µg ml^−1^) and pseudoephedrine (PEPH, 100 µg ml^−1^).

## 3. Results and discussion

### 3.1. Synthesis of 6-monoacetylmorphine–bovine serum albumin conjugate

**Figure 6.**
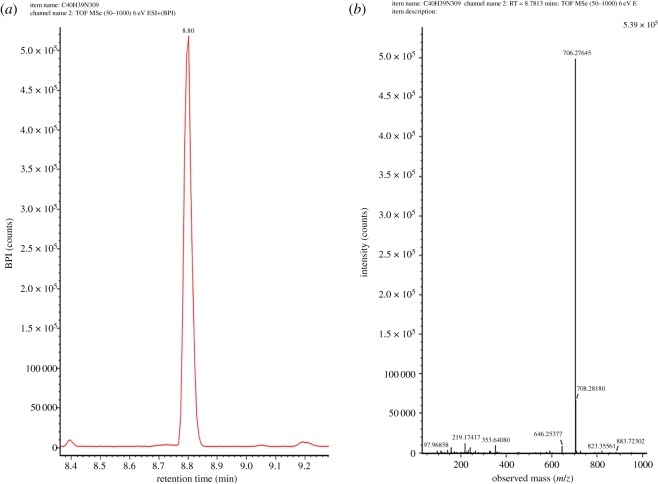
Confirmation of activated 6-MAM by a Waters® Vion IMS Q-Tof mass spectrometer. (*a*) Chromatogram. (*b*) Spectrum.

NC membranes are usually first coated with a carrier protein prior to conjugation of antibody. Linkers are used to maintain structural specificity. Here, an NHS ester group was first added to the 6-MAM molecule as a linker for the carrier protein. This was validated with a Waters® Vion IMS Q-Tof mass spectrometer. We found a broad peak in the ultra-performance liquid chromatography (UPLC) chromatograms of activated 6-MAM at 8.8 min with an *m/z* of 706.27645 (figure 6) versus a predicted *m/z* of 706.2758. This indicates that the linker was successfully attached to the 6-MAM. The significance of precise synthesis is self-evident because only the structure is correctly established, and this could lead to pairing with 6-MAM antibodies.

We did not use gradient dimethylsulfoxide dialysis because the product is soluble and the previous conjugation protocol is too complex. BSA had several peaks in the UPLC chromatogram (data not shown) because of the various analogues. This led to a range of conjugation ratios. Therefore, there were various peaks in the UPLC chromatogram corresponding to the different conjugating ratios. The conjugation results could be more effectively confirmed via antigen–antibody pairing than the conjugating ratio.

### 3.2. Types of nitrocellulose membrane selection

NC membranes bind proteins electrostatically via interactions of the strong dipole of the nitrate ester with the strong dipole of the peptide bonds of the protein. The properties including capillary flow rate, signal intensity and background were evaluated because they could influence the final performance of the LFS. Furthermore, the flow rate is given more attention because it could affect the protein adsorption capacity and even the sensitivity. The flow rate of a membrane depends on the aggregate properties of the porous structures such as pore size, pore size distribution and porosity. A larger pore size leads to weaker protein adsorption.

We compared nine NC membranes (table 1). Each test was repeated three times, and the average result was recorded. The LFS results were measured in 3 min, and the best sample flow rate was below 20 s cm^−1^. The signal intensity at the T line was also needed at the normal level. A deeper background colour hampered accuracy. The Millipore membrane HF135 was the best choice for 6-MAM after comprehensive consideration of sample flow rate, signal intensity at the T line and background colour.

**Table 1.** Types of NC membrane selection for oral fluid 6-MAM LFSs.

	sample flow rate (s cm^−1^)	signal intensity at T line	background colour
Millipore			
HF90	12	weak signal	white
HF135	16	normal signal	white
HF180	29	strong signal	deep red
Whatman			
FF120HP	32	strong signal	red
AE100	21	normal signal	white
Sartorius			
CN95	13	weak signal	white
CN150	19	normal signal	white
Pall			
Vivid90	22	normal signal	pale red
Vivid170	20	strong signal	red

### 3.3. The sample pad

The solution for treating the sample pad is very important for the test because it served as the reaction buffer system when the oral fluid samples rehydrate the pad. The solution usually includes a buffer system with proper ionic strength and pH value; some blocking materials and surfactants can accelerate the oral fluid flow rate on the membrane. The sample pad deals with the complexities of the oral fluid's matrix effects and makes it compatible with the NC membrane. Moreover, the buffer system ensures the release of analytes and stabilizes the flow rate because the oral fluid is too viscous.

Four different formulas were considered. The solution formulas are given in table 2. The results indicated that buffer system 4 with the surfactant STANDAPOL ES-1 (S7) gave the best performance at a sample flow rate of 17 s cm^−1^, with a normal signal intensity and a white background. Surfactant S7 is a strong anionic surfactant that offers stronger washing capability than S17 and S9.

**Table 2.** The solution formulae of the sample pad.

	buffer system 1	buffer system 2	buffer system 3	buffer system 4
formula	borax	NaH_2_PO_4_	Tris	Tris
	OHODASURF	Na_2_HPO_4_	cholic acid sodium	STANDAPOL
	ON-870 (S17)	NaCl	salt (CHL)	ES-1 (S7)
	BSA	Tetronic 1307 (S9)	PVP	S9
		BSA	casein Na	BSA
		PVP	S9	PVP
				CHL
				casein Na

### 3.4. Oral fluid collection

Oral fluids are more difficult to collect than urine due to their high viscosity. There are many kinds of oral fluid-collecting devices: cotton swabs, sponges, plastic tubes and cups. Some methods stimulate oral fluids via vinegar, mouthwashes, lozenges, etc. However, such stimulation may change the concentration of analytes in the oral fluid and is more complicated and time-consuming. We finally collected oral fluid directly from the mouth by a sponge adsorbent (ESSENTRA, UK), which is a blend of polymeric fibres with a suitable pore size.

**Figure 7.**
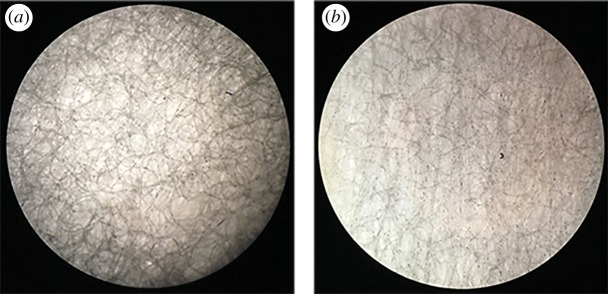
The structures of two sponge pads that were imaged with a microscope (4× objective lens and 10× eye lens). (*a*) K1. (*b*) K2.

Two kinds of sponge adsorbents (K1 and K2) were custom-designed, and the structures of both sponge pads are shown in figure 7. K2 was much looser and regular relative to K1. Both were evaluated by testing the fluid-handling performance including dropping water on the sponge pad, putting the final LFS into the negative potassium phosphate buffer (pH = 7.0) and putting the final LFS into the mouth. The K2 sponge had a twice as fast sample flow rate (average 20 s cm^−1^) than the K1 sponge for water, PBS buffer or real oral fluids. This is a very important issue for oral fluid testing. In conclusion, K2 was chosen for its distinguished performance in dealing with oral fluid samples.

### 3.5. Sensitivity and specificity

**Figure 8.**
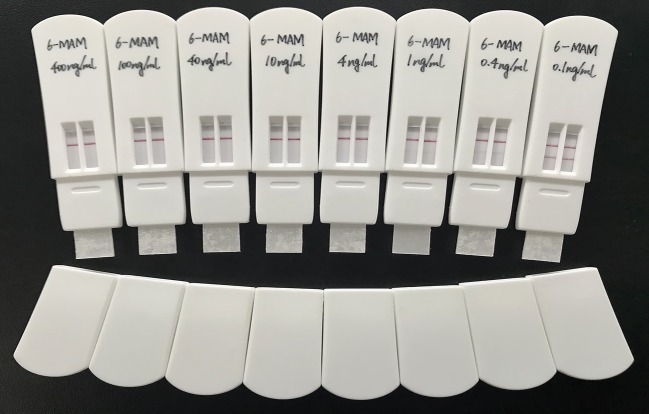
Sensitivity experiments of the LFS. There are testing sample remarks at the top of the strips.

**Figure 9.**
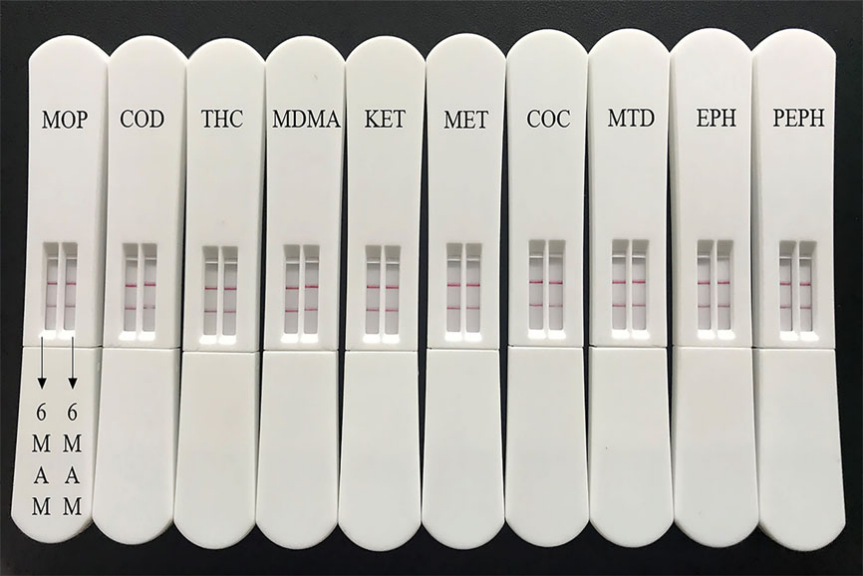
Specificity experiments for the LFSs. At the top of the strips, there are testing sample remarks about the kinds and concentrations including MOP (100 µg  ml^−1^), COD (100 µg ml^−1^), THC (10 µg ml^−1^), MDMA (100 µg ml^−1^), KET (100 µg ml^−1^), MET (100 µg ml^−1^), COC (100 µg ml^−1^), MTD (100 µg ml^−1^), EPH (100 µg ml^−1^) and PEPH (100 µg ml^−1^).

Small molecules are usually detected via a competitive assay in LFSs. Here, there is no signal (red line) in the T line. This represented a concentration of 6-MAM in the sample that is above the cut-off value. The sensitivity for oral fluid testing should be much higher than the urine test because of the low concentration of drug metabolites in oral fluid. Here, we successfully made a qualitative LFS for 6-MAM with a sensitivity of 4 ng ml^−1^, which meets the requirements for general oral fluid detection limits [28,29]. The results are shown in figure 8. In addition, figure 9 shows specificity versus the commonly abused drugs. The 6-MAM LFS was specific to 6-MAM with no cross-reaction especially with morphine or codeine.

## 4. Conclusion

**Figure 10.**
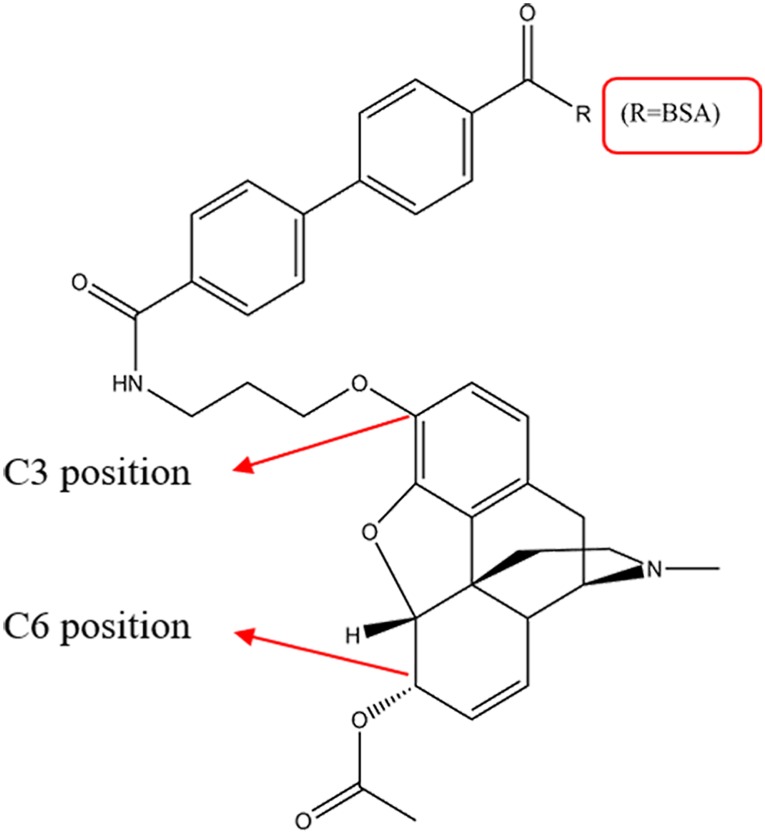
The structure of 6-MAM–BSA.

The 6-MAM is the specific metabolite of heroin. We report here an LFA for 6-MAM via a special conjugate paired with a specific antibody. We made a conjugate that linked 6-MAM to the carrier protein via an NHS ester group at the C3 position (figure 10). Here, the antibody identified the acetyl group of 6-MAM. This is a prerequisite for specificity for 6-MAM. Finally, we made a very sensitive LFS test with no cross-reaction with 10 commonly abused drugs including morphine and codeine. We identified the proper NC membranes, sample pad, pore size and sponge adsorbent to make a test that uses oral fluids at the point of care.

With the above advantages, the 6-MAM LFS for oral fluid sample could be applied to both research and industry uses. It could help the police save manpower and time/costs in preliminary screening. Oral fluids are convenient and less invasive and are suitable for traffic screening. In conclusion, an oral fluid LFS for heroin abuse aimed at 6-MAM is a promising product to combat drugged driving.

## Data accessibility

Data are deposited in the Dryad Digital Repository: https://doi.org/10.5061/dryad.8r36rp3 [34].
